# A Chinese Herbal Formula Ameliorates Pulmonary Fibrosis by Inhibiting Oxidative Stress via Upregulating Nrf2

**DOI:** 10.3389/fphar.2018.00628

**Published:** 2018-06-12

**Authors:** Yunping Bai, Jiansheng Li, Peng Zhao, Ya Li, Meng Li, Suxiang Feng, Yanqin Qin, Yange Tian, Tiqiang Zhou

**Affiliations:** ^1^Henan Key Laboratory of Chinese Medicine for Respiratory Disease, Henan University of Chinese Medicine, Zhengzhou, China; ^2^Collaborative Innovation Center for Respiratory Disease Diagnosis and Treatment – Chinese Medicine Development of Henan Province, Zhengzhou, China; ^3^Institute for Respiratory Diseases, The First Affiliated Hospital, Henan University of Chinese Medicine, Zhengzhou, China; ^4^Dongzhimen Hospital, Beijing University of Chinese Medicine, Beijing, China

**Keywords:** Jinshui Huanxian formula, pulmonary fibrosis, fibroblast, oxidative stress, Nrf2

## Abstract

This study aimed to explore the protective effects of a Chinese herbal formula, Jinshui Huanxian formula (JHF), on experimental pulmonary fibrosis and its underlying mechanisms. After being treated with single dose of bleomycin (5 mg/kg) intratracheally, rats were orally administered with JHF and pirfenidone from day 1 to 42, then sacrificed at 7, 14, 28, or 42 days post-bleomycin instillation. JHF ameliorated bleomycin-induced pathological changes, collagen deposition in the rat lung and recovered pulmonary function at different days post-bleomycin instillation. In lungs of JHF-treated rats, the levels of total superoxide dismutase, catalase and glutathione were higher, and myeloperoxidase and methane dicarboxylic aldehyde were lower than those in vehicle-treated rats, respectively. Additionally, JHF inhibited the expression of NADPH oxidase 4 (NOX4) and increased the Nuclear Factor Erythroid 2-Related Factor 2 (Nrf2) in lung tissues. *In vitro*, JHF and ruscogenin, a compound of Ophiopogonis Radix contained in JHF, significantly inhibited transforming growth factor β1 (TGF-β1)-induced differentiation of fibroblasts. Furthermore, JHF markedly decreased the level of reactive oxygen species in TGF-β1-induced fibroblast. In line with this, upregulation of NAD(P)H: quinone oxidoreductase 1 and heme oxygenase 1, and downregulation of NOX4 were found in JHF-treated fibroblast induced by TGF-β1. While on the other hand, Nrf2 siRNA could suppress the JHF-mediated inhibition effect on alpha-smooth muscle actin (α-SMA), and FN1 expression induced by TGF-β_1_ in fibroblasts. These results indicated that JHF performed remarkably therapeutic and long-term effects on pulmonary fibrosis in rat and suppressed the differentiation of fibroblast into myofibroblast through reducing the oxidative response by upregulating Nrf2 signaling. It might provide a new potential natural drug for the treatment of pulmonary fibrosis.

## Introduction

Pulmonary fibrosis (PF) is a chronic, irreversible and fatal lung disease, characterized by irreversible lung structure damage and exuberant extracellular matrix protein deposition (Luppi et al., 2012). Oxidative stress plays the important roles in the inflammation, collagen deposition, and fibrosis of PF. The free radical activity, lipid products and oxidized proteins have been identified in exhaled air, bronchus alveolar lavage fluid, serum and lung of patients with PF (Daniil et al., 2008; Kliment and Oury, 2010; Faner et al., 2012; Cheresh et al., 2013; Matsuzawa et al., 2015). Reactive oxygen species (ROS), as the important product of oxidative stress could promote apoptosis of airway epithelial cells to trigger inflammation and then collagen deposition by regulating cytokines and growth factors expression and reducing the antioxidant defenses (Faner et al., 2012). NADPH oxidase (NOX) is a family of enzymes that are unique in regulating the primary ROS production (Jarman et al., 2014). Many studies report that NOX4 is selectively upregulated in lung of PF patients (Faner et al., 2012), and NOX4 inhibitor could attenuate PF in a rodent disease model (Luppi et al., 2012). Nuclear factor erythroid 2-related factor 2 (Nrf2), the critical regulator of oxidative stress, can promote the expression of Nrf2-dependent antioxidant, and Nrf2 deficiency clearly contributes to the development of PF (Faner et al., 2012). For instance, Nrf2^-/-^ mice show more severe and earlier onset of fibrotic responses to bleomycin with accompanying accumulation of markers for airway repair and fibrosis, high mortality, marked loss of body weight, and increased lung weight. Moreover, Nrf2-dependent antioxidant defense enzymes in the lungs of Nrf2^-/-^ mice is decreased, which suggests that these enzymes may contribute to Nrf2-mediated protection against bleomycin-induced lung fibrosis (Cho et al., 2004; Walters et al., 2008). In addition, NOX4-Nrf2 imbalance is identified in lung tissues of human subjects with PF, and restoring NOX4-Nrf2 redox balance in myofibroblasts may be an effective therapeutic strategy (Faner et al., 2012). *N*-acetylcysteine, the glutathione (GSH, an important antioxidant molecule) precursor, significantly increases the lung GSH levels in bleomycin-induced fibrosis (Giri et al., 1988; Hagiwara et al., 2000), also increases the percentage of predicted vital capacity and extends the 6 min walking test distance in PF patients (Sun et al., 2016). Recently, two drugs, pirfenidone (PFD) and nintedanib, are approved worldwide for PF treatment (Raghu et al., 2015). However, none drugs have prospectively shown a survival benefit in many trials (Idiopathic Pulmonary Fibrosis Clinical Research Network et al., 2014; Sun et al., 2016). Therefore, it is urgent to find new drugs for treatment of PF.

According to the Chinese medicinal theories and clinical experience, Jinshui Huanxian formula (JHF) was constructed and widely used to prevent PF. The clinical practice showed that JHF exerted extensive pharmacological effects on PF, including alleviating the clinical symptoms, slowing the disease progression, and enhancing live quality. JHF is composed of 11 medicinal herbs, including Ginseng Radix et Rhizoma, Ophiopogonis Radix, Rehmanniae Radix, Trichosanthis Fructus, Fritillariae Thunbergii Bulbus, Moutan Cortex, Epimedii Herba, Ginkgo Semen, Pulsatillae Radix, Coicis Semen, Citri Reticulatae Pericarpium. Many of these herbal drugs have exerted extensive anti-fibrosis and anti-oxidant effect. For example, ginsenoside Rg1 (Ginseng Radix et Rhizoma extract) increases the activities of antioxidant enzymes such as superoxide dismutase (SOD), GSH-Px and catalase (CAT), reduces methane dicarboxylic aldehyde (MDA) levels, and exerts anti-fibrosis effect via promoting Nrf2 (Li et al., 2014). Rehmanniae Radix attenuates fibrosis by downregulating the expressions of transforming growth factor β_1_ (TGF-β_1_), alpha-smooth muscle actin (α-SMA) and Collagen-I (COL-I) (Liu et al., 2015). However, the details about the anti-PF mechanisms of JHF remains poorly understood.

In the light of the association of PF pathogenesis with oxidative stress, we speculated that JHF might have therapeutic effects in PF by weakening oxidative stress. Here, a rat model of bleomycin-induced PF was applied to investigate the anti-PF and anti-oxidative stress effect of JHF. In addition, we investigated the effect of JHF on the TGF-β_1_-induced differentiation of fibroblasts *in vitro* and clarified the anti-oxidative mechanisms of JHF by activating Nrf2. The results of this study may explore the potential mechanisms of JHF and provide the basis for the clinical treatment of PF.

## Materials and Methods

### Chemicals, Herbal Medicines

Jinshui Huanxian formula was prepared by the Pharmaceutical Department in Henan University of Chinese Medicine. JHF consists of 11 medicinal herbs, including Ginseng Radix et Rhizoma (Ren Shen in Chinese), Ophiopogonis Radix (Mai Dong in Chinese), Rehmanniae Radix (Shu Di in Chinese), etc. All herbs were water- or ethanol-extracted and made into dry extract, ultimately. Each 1 g dry extract contains 3.13 g of raw herbs. Bleomycin hydrochloride was obtained from the Nippon Kayaku Co. Ltd. (lot 650427). PFD capsules were obtained from the Beijing Kangdini Pharmaceutical Co. Ltd. (lot 150603) (Beijing, China). Ruscogenin was obtained from Chengdu Must Bio-Technology Co. Ltd. (MUST-16061703).

### Animals

Sprague-Dawley rats (weight 180–220 g) were obtained from the Experimental Animal Center of Henan Province (Zhengzhou, China). The rats were raised under controlled temperature (26–28°C), humidity (50 ± 10%) and daily light intensity (12 h light/12 h dark cycle), were fed with standard laboratory food and water *ad libitum*. This study was carried out in accordance with the recommendations of the ‘Care and Use of Laboratory Animals of guidelines, the First Affiliated Hospital of Henan University of Chinese Medicine’ and ‘Experimental Animal Care and Ethics Committee of the First Affiliated Hospital, Henan University of Chinese Medicine.’ The protocol was approved by the ‘Experimental Animal Care and Ethics Committee of the First Affiliated Hospital, Henan University of Chinese Medicine.’

### Preparation of JHF-Containing Serum

One hundred Sprague-Dawley rats were randomly divided into two groups. Rats in Group 1 were orally administrated with JHF (0.864 g/ml) at a dose of 10 ml/kg body weight twice a day for 7 days. Rats in Group 2 were orally administrated with an equivalent volume of distilled water. After the final oral administration on day 7, all rats were anesthetized with 4% chloral hydrate. Blood samples were collected from the portal vein and then centrifugated at 3500 rpm for 15 min. The serum samples were collected and kept frozen at -80°C.

### Determinations of the Main Chemical Constituents in JHF-Containing Serum

Four milliliters of methanol was added to 1 ml of the serum samples and stirred well. The mixture was centrifuged at 12,000 rpm for 15 min at 4°C, and the supernatant was evaporated to dryness at 40°C under gentle streams of nitrogen. The dry residue was reconstituted by 100 μl of methanol, then centrifuged again at 12,000 rpm for 15 min at 4°C, and 5 μl of the supernatant was used for LC-MS/MS analysis. An Ultimate 3000 UPLC system coupled with an Q-Exactive mass spectrometer (Thermo Scientific, United States) with a heated electrospray ionization source, was used for detecting the compounds contained in drug-containing serum. Chromatographic separation was performed on a Hypersil GOLD C18 column (4.6 mm × 250 mm, 5 μm) (Thermo Scientific, United States) at a flow rate of 1.0 ml/min at 30°C. The mobile phases consisted of 0.1% formic acid in water (A) and acetonitrile (B). The gradient elution program was as follows: 0–5 min, 5% B; 5–25 min, 5–80% B; 25–35 min, 80–100% B; 35–45 min, 100% B; 45–50 min, 5% B. Mass spectrometry detection was carried out in fast polarity-switching mode. Parameters of the ion source were as follows: spray voltage, 3.5 KV (+) and 2.8 KV (-); capillary temperature, 350°C; flow rate of the sheath gas, 35 arbitrary units; flow rate of the auxiliary gas, 10 arbitrary units. The mass scan range was from 150 to 1,500 m/z, and the resolution was at 70000.

### Bleomycin-Induced PF in Rats and Drug Administration

Sprague-Dawley rats were randomly divided into four groups (control group, Model group, JHF group, and PFD group). Rats were anesthetized with 10% chloral hydrate (3.0 ml/kg) by intraperitoneal injection. Then, 5 mg/kg of bleomycin in sterile 0.9% NaCl was intratracheally injected into the rats of Model group, JHF group and PFD group (Degryse et al., 2010). The rats of control group were given the same volume of sterile saline instead of bleomycin. From day 1 after intratracheal injection, the rats in Control and Model groups were intragastrically administrated with normal saline, JHF group administrated with JHF (10.8 g/kg body weight), PFD group administrated with PFD Capsules (50 mg/kg body weight), once a day from day 1 to 42. On day 7, 14, 28, and 42, eight rats in each group were sacrificed, then their lung tissues were harvested. Lung coefficient was determined by lung weight (mg) versus body weight (g).

### Forced Vital Capacity

Forced vital capacity (FVC) was determined with a computer controlled pulmonary function test system (BUXCO, DSI, St. Paul, MN, United States) before rats of each group were sacrificed. After anesthetized and endotracheally intubated, rats were placed in the sealed chamber and connected to the device via the intubation, and the respiratory data was acquired with a pressure volume transducer and presented with FlexiVent software (BUXCO, DSI, St. Paul, MN, United States).

### Collagen Determination and Histologic Grading of Fibrosis

Lung tissues were fixed in 10% phosphate-buffered formalin, embedded in paraffin, sectioned at 5 μm, and stained with hematoxylin-eosin solution (Solarbio, Beijing, China), and Masson’s Trichrome stain kit (Solarbio, Beijing, China) to determine the collagen distribution. Fibrosis was quantified using the entire lung by the Ashcroft scoring system (Ashcroft et al., 1988).

### Immunohistochemical Analysis

Sections were block with 5% bull serum albumin (BSA) for 20 min and incubated with antibodies against TGF-β_1_ (1:150 dilution, Bioss, Beijing, China), α-SMA (1:100 dilution, Bioss, Beijing, China), COL-I (1:150 dilution, Bioss, Beijing, China), COL-III (1:120 dilution, Bioss, Beijing, China), at 4°C for 12 h, followed by incubation with goat anti-rabbit immunoglobulin G (ZSGB-BIO, Beijing, China) at 25°C for 2 h, then the sections were counterstained with hematoxylin. The expressions of up-mentioned proteins were observed with a Leica microscope, and images were collected for semi-quantitative analysis achieved by Image-Pro Plus 6.0 professional image acquisition and analysis system (Media Cybernetics, Rockville, MD, United States).

### Hydroxyproline Content Assay

The hydroxyproline content in the lung was determined by the spectrophotometric method according to the hydroxyproline assay kit instruction (Jiancheng, Nanjing, Jiangsu, China). The data are expressed as micrograms of hydroxyproline per gram wet lung weight (μg/g tissue).

### Analysis of T-SOD, CAT, GSH, MDA, and MPO in Serum and Lung Tissues

Activity of T-SOD, CAT and MPO, MDA and GSH in serum and lung tissues were measured by hydroxylamine and thiobarbituric acid methods, respectively, according to the manufacturer’s protocol (Jiancheng, Nanjing, Jiangsu, China).

### Cell Culture and Transfection

The human lung fibroblast MRC-5 cells and the mouse fibroblast NIH-3T3 cells (obtained from Shanghai Institutes for Biological Sciences, China) were cultured in MEM (Boster Biological Technology Co., Wuhan, China) containing 10% FBS, and maintained in a humidified atmosphere with 5% CO_2_ at 37°C. when cells were grown to 80–90%, cells were plated on dishes at a density of 2 × 10^5^ cells/ml and incubated in 5% CO_2_–95% air for 24 h. Then they were transfected with Nrf2 siRNA (50 nM) (RIB BIO, Guangzhou, China) for 24 h. Transfection was performed in serum-free medium using Lipofectamine 2000 (Invitrogen, Carlsbad, CA, United States) according to the manufacturer’s instructions. Next day, cells were treated with TGF-β_1_ (2.5 ng/ml) and JHF-containing serum (2.5%, 5%) in MEM and harvested 24 h later.

### RNA Isolation and Real-Time PCR Analysis

The primers were designed and synthesized by Genscript Biotech Co. Ltd. (Nanjing, China). Total RNA was extracted by using TRIzol reagent (Ambion, Foster City, CA, United States) according to the instructions. Concentration and integrity of total RNA were verified by a NanoDrop 2000 nano-spectrophotometer (Thermo, Waltham, MA, United States). Reverse transcription (RT) was proceeded by using SuperScript^®^ III First-Strand Synthesis Super Mix for qRT-PCR Kit (Invitrogen, Carlsbad, CA, United States), and real-time PCR reactions were performed using Platinum^®^ SYBR^®^ Green qPCR SuperMix-UDG with ROX Kit (Invitrogen, Carlsbad, CA, United States). The reaction systems were prepared following the instructions of the kits and reacted on an Applied Biosystems 7500/7500 Fast Real-Time PCR System (AB, Foster City, CA, United States). The initial enzyme activation step was at 95°C for 2 min, followed by 40 cycles of 95°C for 15 s, 60°C for 30 s. At the end of PCR, to evaluate specific amplification of the target genes, melting curve ranging from 60 to 95°C were also included in each run.

### Western Blotting

The samples were homogenized in RIPA buffer containing PMSF (Solarbio, Beijing, China) for 30 min. After centrifugation (12,000 r/min, 5 min at 4°C), the total protein was collected, and the protein concentrations were detected by BCA method. Then protein denaturalization was performed at 100°C for 10 min. Proteins in the supernatant were separated by SDS-PAGE on a 10% gel and then transferred to PVDF membranes (Millipore, Bedford, MA, United States). The blotted membranes were blocked with 5% non-fat dry milk. Then blotted membranes were incubated with anti-NOX4, Nrf2 (CST, MA, United States) and GADPH (Proteintech, Wuhan, China) antibodies. After being washed with TBST three times (10 min per time), the blots were visualized with the Super ECL Plus reagent (Solarbio, Beijing, China), and were scanned and quantified by Chemi Doc^TM^ MP System (Bio-Rad, Hercules, CA, United States).

### Immunofluorescence Analysis

Cells were plated in 12-well plates for 24 h and then treated with TGF-β_1_ (0, 1.25, 2.5, 5, 10 ng/ml) for 24 h. Cells were washed with PBS, fixed with 4% formaldehyde for 15 min, followed by permeabilization with 0.1% Triton X-100 for 20 min. Then cells were blocked with 5% normal goat serum in PBS followed by incubation with primary antibody over night at 4°C. Thereafter, cells were incubated with a secondary antibody for 1 h. Cells were washed and mounted in ProLong Gold antifade reagent with DAPI. Cells were visualized using confocal laser scanning microscopy (OLYMPUS FV1000, Japan).

### The Protein Levels of FN and COL-I

The expression levels of FN and COL-I in the supernatant of NIH-3T3 cells were determined by Mouse FN ELISA Kit (Boster, Wuhan, China) and Mouse COL-I ELISA Kit (Elabscience, Wuhan, China) according to the manufacturer’s instructions. The colorimetric reaction was measured at 450 nm.

### Intracellular ROS Detection

Reactive oxygen species level was measured by using ROS Assay Kit (Solarbio, Beijing, China). Cells were rinsed with PBS, then incubated with DCFH-DA (10 μM) at 37°C for 1 h in the dark. The cells were rinsed with PBS again. Intracellular ROS was analyzed by FACS scan flow cytometer (Beckman, United States).

### Statistical Analysis

Data are expressed as mean ± SEM. Statistical differences between the groups were performed by one-way ANOVA. Differences were considered to be significant at *P*-values < 0.05.

## Results

### JHF Ameliorated Bleomycin-Induced Lung Fibrosis

In order to clear the therapeutic effect of JHF, JHF were administrated to bleomycin-induced-PF rats. Because PFD have been approved for the treatment of PF (Raghu et al., 2015), we used it as a positive control drug. JHF and PFD, were administrated to rats on 1 day after injecting bleomycin. The anti-PF effect of JHF and PFD were evaluated by assessing changes of FVC, lung coefficient, and collagen content.

**FIGURE 1:**
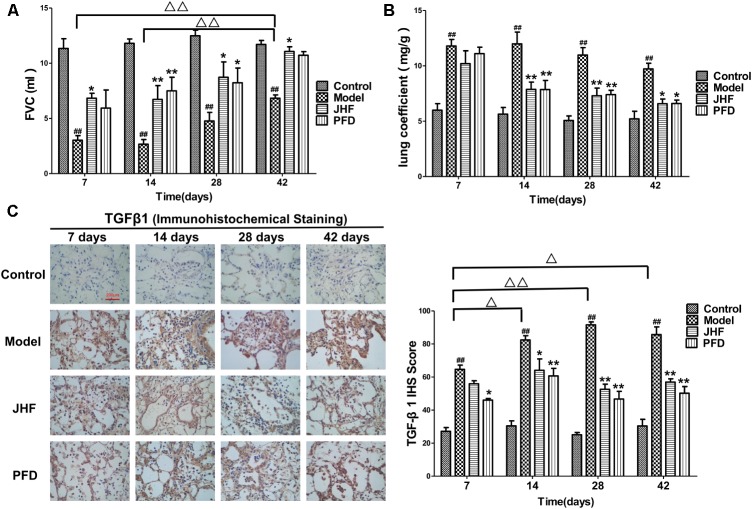
Jinshui Huanxian formula (JHF) inhibited bleomycin-induced the decline of FVC, increase of Lung coefficient and up-expression of TGF-β1. **(A)** FVC. **(B)** Lung coefficient. **(C)** Immunohistochemical staining of TGF-β1 in rat lung tissue, scale bars: 200 μm. Values represented as mean ± SEM. ^##^*P* < 0.01, versus control group. ^∗∗^*P* < 0.01, ^∗^*P* < 0.05, versus model group. ^Δ Δ^ *P* < 0.01, ^Δ^ *P* < 0.05, versus matched time point model group.

Jinshui Huanxian formula inhibited the decline of FVC caused by bleomycin. As shown in **Figure 1A**, FVC was markedly decreased in rats of model group on day 7, 14, 28, and 42, and had a recovery in spite of below baseline on day 42. While the decreases of FVC were significantly inhibited by JHF and PFD treatment. There was no significant difference between the inhibition effect of JHF and PFD.

Jinshui Huanxian formula suppressed the increases of lung coefficient and TGF-β1 expression induced by bleomycin. As shown in **Figure 1B**, lung coefficient was rapidly increased on day 7 and 14 after bleomycin administration, which was markedly decreased by JHF and PFD treatments on day 14. As shown in **Figure 1C**, the expression of TGF-β_1_ of bleomycin-induced PF was upregulated compared with that in the control group. Compared with bleomycin-induced PF model, the expression of TGF-β_1_ was markedly inhibited by JHF and PFD treatments from day 14 to 42.

**FIGURE 2:**
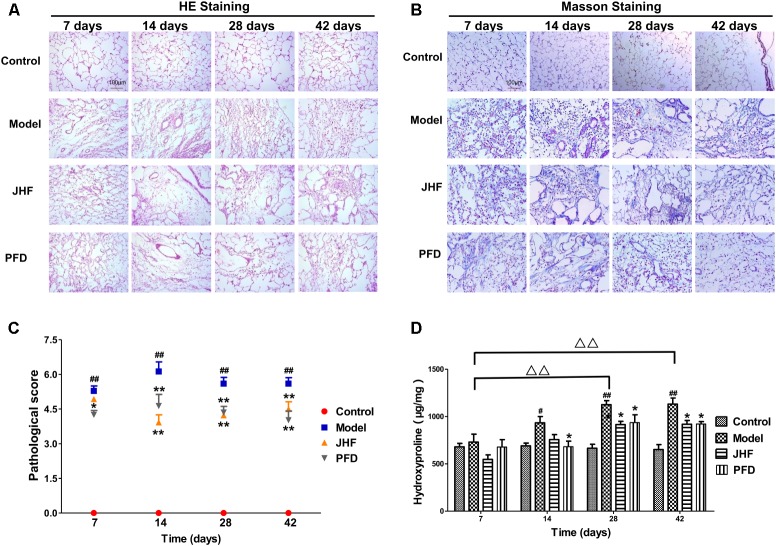
The therapeutic effect of Jinshui Huanxian formula (JHF) on bleomycin-induced PF rats. **(A)** Hematoxylin and eosin stained, scale bars: 100 μm. **(B)** Masson trichrome stained, scale bars: 100 μm. **(C)** Ashcroft score, **(D)** Hydroxyproline. Values represented as mean ± SEM.^##^*P* < 0.01, ^#^*P* < 0.05, versus control group. ^∗∗^*P* < 0.01, ^∗^*P* < 0.05 versus model group. ^Δ Δ^ *P* < 0.01, ^Δ^ *P* < 0.05, versus matched time point model group.

**FIGURE 3:**
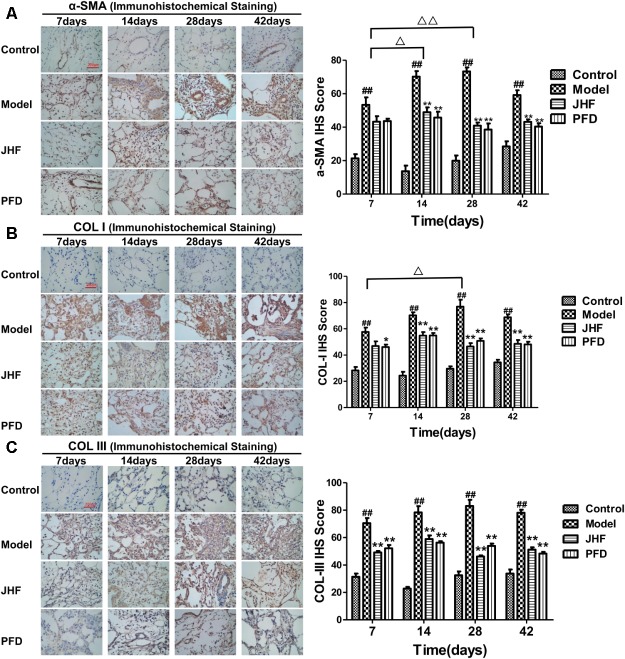
Jinshui Huanxian formula (JHF) suppressed α-SMA, COL-I, and COL-III expression in bleomycin-induced PF rats. Immunohistochemical analysis of α-SMA **(A)**, COL-I **(B)**, and COL-III **(C)**, scale bars: 200 μm. Values represented as mean ± SEM. ^##^*P* < 0.01, versus control group. ^∗∗^*P* < 0.01, ^∗^*P* < 0.05, versus model group. ^Δ Δ^ *P* < 0.01, ^Δ^ *P* < 0.05, ANOVA versus matched time point model group.

Jinshui Huanxian formula reduced the expression of collagen induced by bleomycin in the lung. As shown in **Figures 2A–C**, gathering of neutrophil and macrophage, and thickening on alveolar walls were observed in lung parenchyma of model rats from day 7 to 14. Meanwhile, fibrosis foci marked with disruption of the alveolar unit and deposition of collagen were also observed on day 7, aggravated from day 14. On day 28 and 42, collagen accumulation in the fibrosis foci was significantly increased. Both JHF and PFD treatments significantly suppressed the histopathological changes of lung tissues by reducing inflammatory cells infiltration and collagen accumulation from 7 to 42 days. As shown in **Figure 2D**, hydroxyproline was markedly decreased from day 28 to 42 after the treatment of JHF and PFD. There was no significant difference between the inhibition effect of JHF and PFD. As shown in **Figures 3A–C**, the expression levels of α-SMA, COL-I, Collagen-III (COL-III) in the rats of model group were increased compared with that in the control group. While these increases were markedly inhibited by JHF and PFD treatments from day 14 to 42. Taken together, the results showed that JHF prevented bleomycin-induced pulmonary damage and fibrosis, improved respiratory function.

### JHF Reduced Bleomycin-Induced Oxidative Stress

Oxidative stress contributes to the development of PF. To confirm the effect of JHF on the oxidative stress, we measured the expression of antioxidants and oxidants in lung tissue and serum.

**FIGURE 4:**
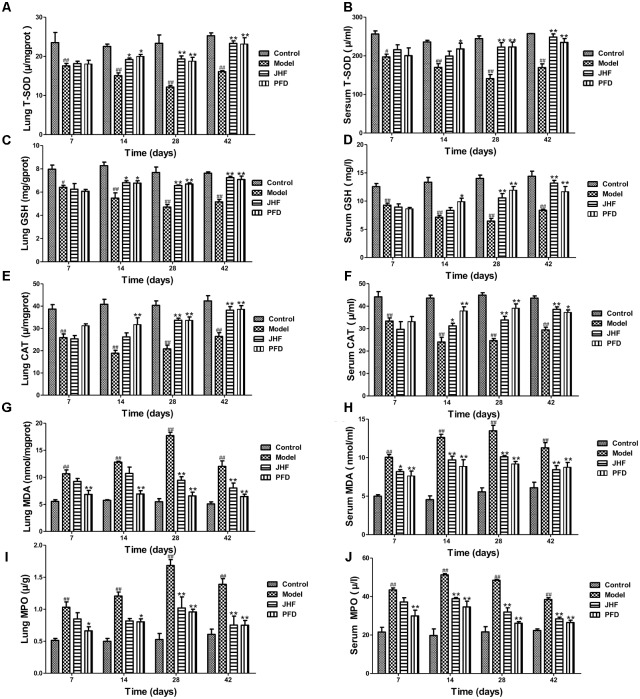
Jinshui Huanxian formula (JHF) ameliorated bleomycin-induced oxidative stress in rat lung tissue and serum. **(A,B)** T-SOD activity, **(C,D)** the level of GSH content, **(E,F)** CAT activity. **(G,H)** MDA level, **(I,J)** MPO activity. Values represented as mean ± SEM. ^##^*P* < 0.01, ^#^*P* < 0.05, versus control group. ^∗∗^*P* < 0.01, ^∗^*P* < 0.05, vs. model group.

As shown in **Figures 4A–F**, T-SOD, CAT activity and GSH content were markedly decreased in rat lung and serum of model group, which were markedly inhibited by JHF and PFD treatments from day 14 to 28. On the other hand, MDA expression was rapidly increased after bleomycin treatment, and at peak on day 28, with a slight decrease on day 42 in both lung tissue and serum. MDA level was markedly decreased in the serum after 7 days of JHF and PFD treatment, and decreased in lung tissue after 28 days of JHF and PFD treatment (**Figures 4G,H**). During day 7 to 42, the activity of MPO was increased in bleomycin-treated rat. Similarly, administration of JHF or PFD significantly decreased MPO activity in lung tissue and serum (**Figures 4I,J**). These results suggested that bleomycin-treated rats exhibited the decrease of enzymatic antioxidants and increase of lipid peroxide, while JHF treatment could suppressed the changes caused by bleomycin.

**FIGURE 5:**
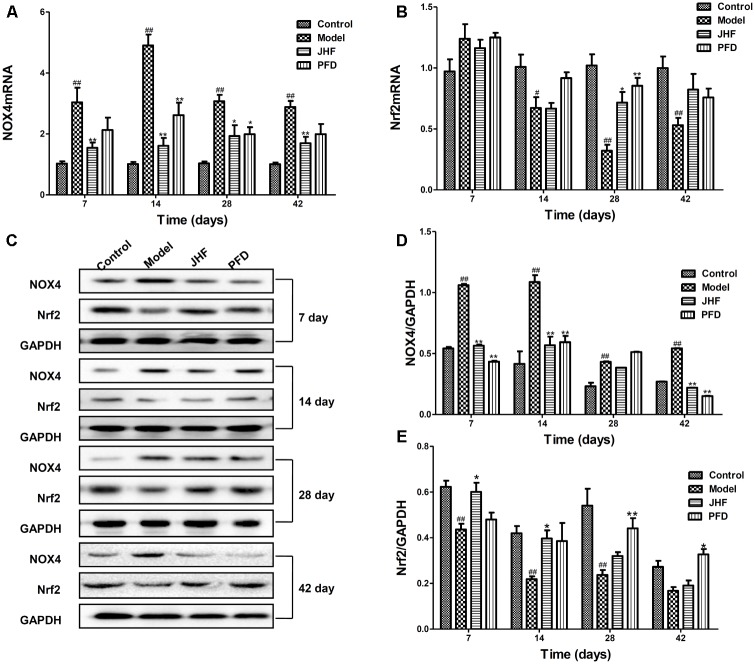
Jinshui Huanxian formula (JHF) inhibited expression of NOX4 and increased expression of Nrf2 mRNA and protein on PF rats. **(A,B)** The expressions of NOX4 and Nrf2 mRNA in lung tissue. **(C–E)** The expression of NOX4 and Nrf2 protein in lung tissue. Values represented as mean ± SEM. ^##^*P* < 0.01, ^#^*P* < 0.05, versus control group. ^∗∗^*P* < 0.01, ^∗^*P* < 0.05, versus model group.

To explore the antioxidant mechanisms of JHF, we investigated the mRNA and protein levels of NOX4 and Nrf2 in lung tissues of PF rats. As shown in **Figures 5A,B**, compared to that in control group, NOX4 mRNA level was markedly upregulated from day 7 to 42 and Nrf2 mRNA level was markedly decreased from day 14 to 42 in lung tissue of bleomycin-treated rats. While, JHF could downregulate the NOX4 mRNA levels from day 7 to 42, upregulate the Nrf2 mRNA levels on day 28. Similarly, PFD could upregulate the Nrf2 mRNA on day 28 and downregulate the NOX4 mRNA level on day 14 and 28. Additionally, the Nrf2 protein level was markedly decreased from day 7 to 28, and NOX4 protein level was markedly upregulated from day 7 to 42 in lung tissue of bleomycin-treated rat. JHF could upregulate the Nrf2 on day 7 and 14 and downregulate the NOX4 expression levels on day 7, 14, and 42. PFD could upregulate the Nrf2 on day 28 and 42 and downregulate the NOX4 expression levels on day 7, 14, and 42 (**Figures 5C–E**). These data indicated that JHF protected against bleomycin-induced oxidative stress by inhibiting peroxide and enhancing antioxidant ability which was probably associated with the upregulation of Nrf2 and downregulation of NOX4.

### JHF Inhibited TGF-β_1_-Induced Differentiation of Fibroblasts

**FIGURE 6:**
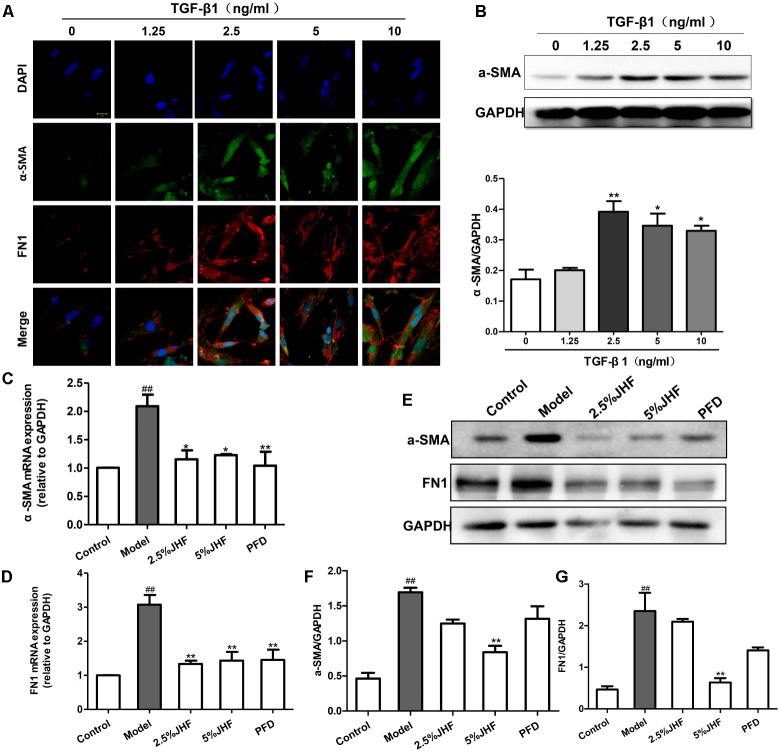
Jinshui Huanxian formula (JHF) inhibited TGF-β1-induced differentiation of MRC-5 cells. **(A)** Immunofluorescence analysis of α-SMA and FN1 expression in TGF-β1-treated, α-SMA in green, FN1 in red and DAPI in blue, scale bars: 20 μm. Values represented as mean ± SEM. ^∗∗^*P* < 0.01, ^∗^*P* < 0.05, versus control group. **(B)** The protein level of α-SMA in TGF-β-treated fibroblasts. Values represented as mean ± SEM. ^##^*P* < 0.01, versus control group. ^∗∗^*P* < 0.01, ^∗^*P* < 0.05, versus model group. **(C–G)** The effect of JHF on mRNA and protein levels of α-SMA and FN1 in TGF-β1-induced fibroblasts. Values represented as mean ± SEM. ^##^*P* < 0.01, versus control group. ^∗∗^*P* < 0.01, versus model group.

**FIGURE 7:**
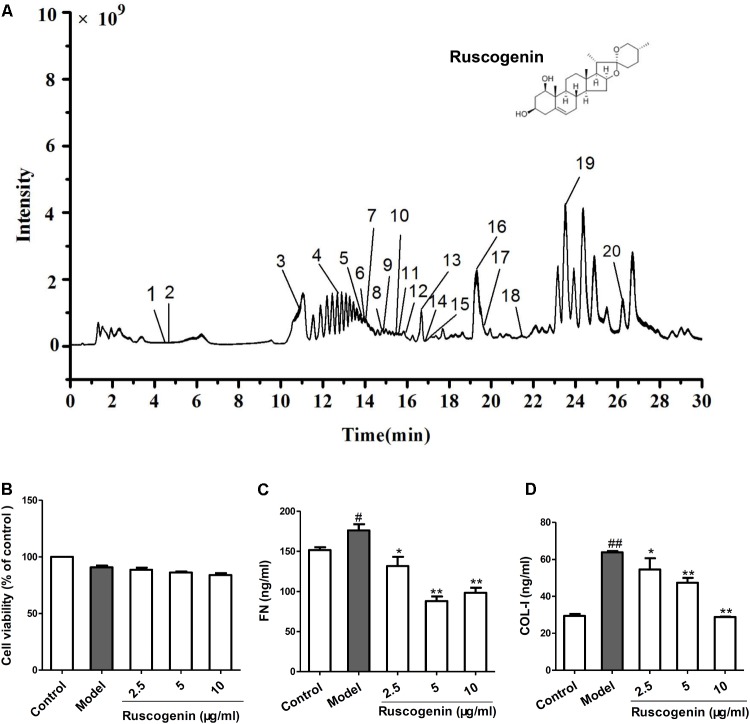
Total ion chromatogram of Jinshui Huanxian formula (JHF)-containing serum, and the effect of ruscogenin on the differentiation of fibroblast. **(A)** The 20 compounds were identified in JHF-containing serum: 1, Catalpol; 2, geniposide; 3, leonuride; 4, bilobalide; 5, Peimine; 6, hesperidin; 7, Peiminine; 8, Ginsenoside Re; 9, Ginkgolide B; 10, Ginkgolide A; 11, Anemoside B4; 12, Icraiin; 13, Ophiopogonin D; 14, Ginsenoside Rb1; 15, Ginsenoside Rc; 16, Nobiletin; 17, Methylophiopogonanone B; 18, Paeonol; 19, Ruscogenin; 20, 3,29-Dibenzoyl rarounitriol. **(B)** The effect of ruscogenin on NIH-3T3 cell viability was detected by MTT analysis. **(C,D)** The NIH-3T3 cells were treated with TGF-β1 (7.5 ng/ml) and different concentrations of ruscogenin (2.5, 5, 10 μg/ml) for 24 h. Then the protein levels of FN and COL-I in the supernatant of NIH-3T3 cells were detected. Values represented as mean ± SEM. ^##^*P* < 0.01, versus control group. ^∗∗^*P* < 0.01, ^∗^*P* < 0.05, versus model group.

To investigate the mechanism of JHF in fibroblast differentiation, we treated TGF-β_1_-induced MRC-5 cell with JHF-containing serum. Firstly, we exposed MRC-5 cell to different concentrations of TGF-β_1_ (1.25, 2.5, 5, and 10 ng/ml), and found that 2.5 ng/ml of TGF-β_1_ could significantly upregulate the expression of α-SMA and FN1 in MRC-5 cells (**Figures 6A,B**). We therefore selected TGF-β_1_ concentrations of 2.5 ng/ml for MRC-5 cells in all subsequent experiments. As shown in **Figures 6A–G**, TGF-β_1_ (2.5 ng/ml for 24 h) significantly upregulated α-SMA and FN1 mRNA and protein levels, while JHF and PFD could markedly inhibit the increases. These data showed that JHF could clearly suppress differentiation of fibroblast. In addition, we identified 20 compounds from JHF-containing serum, including catalpol, geniposide, leonuride, bilobalide, Peimine, hesperidin, Peiminine, Ginsenoside Re, Ginkgolide B, Ginkgolide A, Anemoside B4, Icraiin, Ophiopogonin D, Ginsenoside Rb1, Ginsenoside Rc, Nobiletin, Methylophiopogonanone B, Paeonol, Ruscogenin, and 3,29-Dibenzoyl rarounitriol. (**Figure 7A**). Then, we test the effect of ruscogenin, presented in JHF-containing serum, on differentiation of fibroblast. As shown in **Figures 7B–D**, we found ruscogenin had no effect on the cell viability, while significant inhibited TGF-β1-induced increase of FN and COL-I in NIH-3T3 cell. These results suggested that these components included in JHF might be the anti-fibrosis substances of JHF.

### JHF Suppressed ROS Production in MRC-5 Cells Induced by TGF-β_1_

**FIGURE 8:**
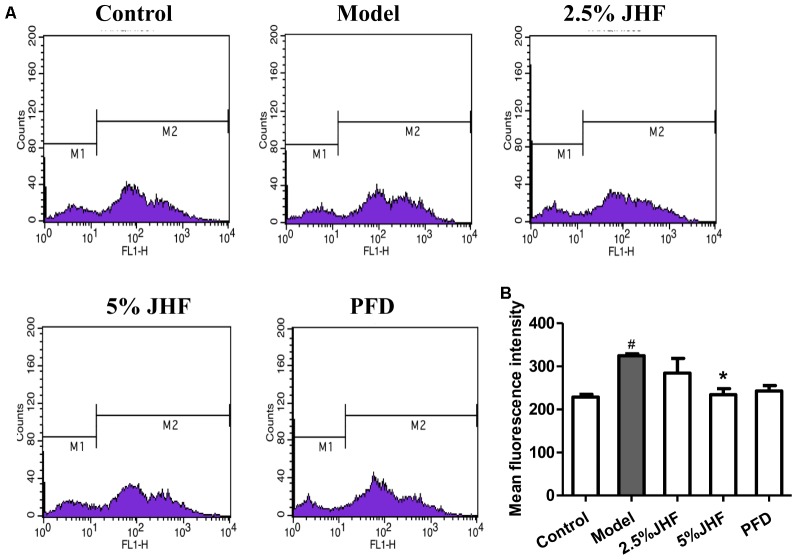
Jinshui Huanxian formula (JHF) reduced the level of ROS in TGF-β1-induced MRC-5 cells. **(A)** After being treated with TGF-β1 JHF and PFD for 24 h, MRC-5 cells were incubated with CM-H2DCFDA (10 μM) for 30 min, fluorescence of DCF was measured by flow cytometry. **(B)** ROS levels in MRC-5 cells were quantified and presented. Values represented as mean ± SEM. ^#^*P* < 0.05, versus control group. ^∗^*P* < 0.05, versus model group.

Reactive oxygen species plays a critical role in the differentiation of fibroblast and activation of myofibroblasts in PF. The high ROS levels have been detected in PF fibroblasts (Bocchino et al., 2010). To investigate the role of ROS in therapeutic effect of JHF, we measured the ROS levels in JHF-treated-MRC-5 cells. As shown in **Figures 8A,B**, the result showed that 5% JHF markedly suppressed intracellular ROS production induced by TGF-β_1_ (2.5 ng/ml for 24 h) in MRC-5 cells. The result suggested that regulating ROS might be play an important role in the therapeutic effect of JHF.

### JHF Suppressed NOX4 and Nrf2 Signaling Pathway

**FIGURE 9:**
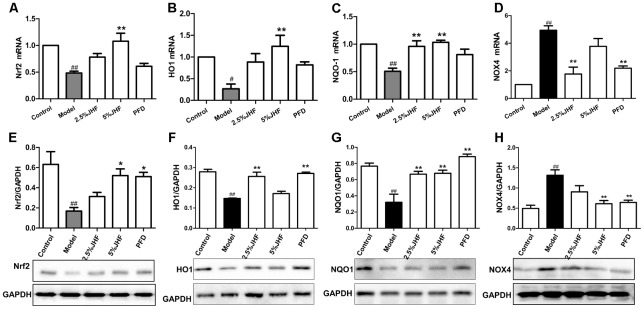
The effect of Jinshui Huanxian formula (JHF) on mRNA and protein levels of Nrf2, HO1, NQO-1, and NOX4 in TGF-β1-induced fibroblasts. **(A–D)** RT-PCR analysis of Nrf2, HO1, NQO-1, and NOX4 mRNA expression in TGF-β1-induced fibroblasts. Reported values are means ± SEM. of the relative fold inductions calculated with the Δ ΔCt method considering control values as the calibrator, i.e., expression level = 1. **(E–H)** Representative Western blot analysis of Nrf2, HO1, NQO-1, and NOX4 protein expression and quantitation with mean values ± SEM. ^##^*P* < 0.01, ^#^*P* < 0.05, versus control group. ^∗∗^*P* < 0.01, ^∗^*P* < 0.05, versus model group.

NADPH oxidase 4 is the critical regulator in the process of ROS production, and Nrf2 is the key transcription factor of antioxidant. Moreover, the up-regulation of NOX4 and down-regulation of Nrf2 have been proven to be involved both in lung tissues and myofibroblasts of PF (Hecker et al., 2009; Hecker et al., 2014). We next assessed whether the imbalance of NOX4-Nrf2 was suppressed by JHF in TGF-β_1_- induced MRC-5 cells. As shown in **Figures 9A–H**, after administration of TGF-β_1_ for 24 h, the mRNA levels of Nrf2 and its downstream gene including heme oxygenase 1 (HO-1) and NAD(P)H: quinone oxidoreductase 1 (NQO1) were significantly decreased, while that of NOX4 was significantly increased. JHF could inhibit these changes induced by TGF-β_1_. Similarly, JHF and PFD could inhibit NOX4 protein expression and increase Nrf2, HO-1, and NQO1 protein expressions.

### Nrf2 siRNA Prevented the Anti-fibrosis Effect of JHF on MRC-5 Cells Induced by TGF-β_1_

**FIGURE 10:**
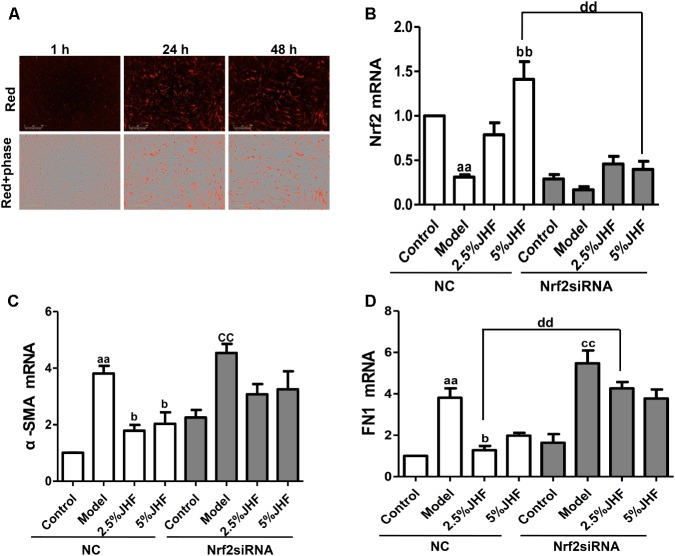
Nrf2 siRNA prevented the anti-fibrosis effect of Jinshui Huanxian formula (JHF) on TGF-β1-induced MRC-5 cells. **(A)** MRC-5 cells were transfected with Cy3-labeled siRNA (red), scale bars: 300 μm. **(B)** The effect of JHF on the mRNA level of Nrf2 in TGF-β1-treated fibroblasts transfected with Nrf2 siRNA. **(C)** The effect of JHF on the mRNA levels of α-SMA, FN1 in TGF-β1-treated fibroblasts transfected with Nrf2 siRNA. Values represented as mean ± SEM. ^aa^*P* < 0.01, versus NC control group. ^bb^*P* < 0.01, ^b^*P* < 0.05, versus NC model group. ^cc^*P* < 0.01, versus Nrf2 siRNA control group.

To further explore whether regulating Nrf2 contributes to the anti-fibrosis effect of JHF, we knocked down the Nrf2 in MRC-5 cells. As shown in **Figures 10A–D**, the results showed that Nrf2 siRNA were successfully delivered into MRC-5 cell and significantly decreased the expression of Nrf2. Moreover, Nrf2 siRNA could prevent the inhibition effect of JHF on α-SMA, FN1 mRNA expression induced by TGF-β_1_ in MRC-5 cells. These results demonstrated that JHF inhibited differentiation of fibroblasts probably through reducing the oxidative response by restoring the balance of Nrf2–NOX4.

## Discussion

Traditional herbal medicine composed of the mixture of various herbs with wide pharmacological activities have been widely used in China and other many countries for thousands years (Isohama et al., 1997). JHF, a traditional herbal formula for PF treatment, was constructed by professor Jiansheng Li, and had been clinically shown beneficial effect on PF by alleviating the clinical symptoms, slowing the disease progression, and enhancing live. In this work, our data showed that JHF suppressed the expression of collagen protein, TGF-β_1_, α-SMA and improved lung coefficient and respiratory function, and subsequently exerted the pharmacological effects in preventing bleomycin-induced pulmonary damage and fibrosis.

Oxidative stress participated in the course of PF pathogenesis (Kinnula et al., 2005). For example, pulmonary inflammatory cells of PF patients generate higher levels of oxidants than those in control patients (Kinnula et al., 2005). Moreover, ROS generated from the mitochondrium causes not only cellular oxidative stress but also apoptosis of alveolar epithelial cells (Kuwano et al., 2003). 8-Isoprostane, a biomarker of oxidative stress, myeloperoxidase and eosinophil cationic protein, are also increased in the bronchoalveolar lavage fluid (BALF) of IPF patients (Montuschi et al., 1998; Kliment and Oury, 2010). The mechanisms by which oxidative stress contributes to pathogenesis may be associated with the alterations in redox signaling. Specifically, the aberrant up-regulation of the ROS-generating enzyme NOX4, coupled with the low-expressed Nrf2, results in a sustained redox imbalance, which promote the activation and differentiation of fibroblasts and make myofibroblast get apoptosis-resistant phenotype (Amara et al., 2010; Hecker et al., 2014). Many data have shown the anti-oxidative effects of TCM on PF, for example, Danggui Buxue Tang and TJ-19 (two different TCM compounds) could attenuate BLM-induced PF via increasing SOD activity and decreasing MDA content (Yang et al., 2010; Zhao et al., 2015). In this study, we demonstrated that JHF treatment elevated the levels of antioxidant including T-SOD, GSH, CAT, and reduced the levels of oxidant index including MDA, MPO in lung and serum of bleomycin-induced PF rat. Moreover, JHF could significantly upregulate the expression of Nrf2 and downregulate NOX4 expression. The data of *in vivo* experiment provided some details of therapeutic mechanism of JHF for treating PF.

Fibroblasts are considered to play a major role in the aberrant ECM turnover of PF. And TGF-β_1_, a critical regulator in the late stages of fibrosis induction (Jiang et al., 2016), can initiate the differentiation of fibroblasts into myofibroblasts (Scotton and Chambers, 2007). In addition, TGF-β_1_ can significantly increase ROS production to promote collagen production by activating differentiation of fibroblasts into myofibroblasts (Liu and Gaston Pravia, 2010; Liu et al., 2012). In the present study, JHF effectively attenuated TGF-β_1_-induced differentiation of fibroblasts into myofibroblasts by reducing the expression levels of FN1 and α-SMA. Furthermore, JHF also weakened the TGF-β_1_-induced oxidative stress by inhibiting endogenous ROS production and the expression of NOX4, and upregulating the expression of Nrf2, NQO1, and HO1. Using siRNA to silence the Nrf2 in MRC-5 cells, we found knockdown of Nrf2 significantly reduced the inhibition effects of JHF on the TGF-β_1_-induced differentiation of fibroblasts into myofibroblasts. Therefore, these results demonstrated that JHF could inhibit the TGF-β_1_-induced differentiation of fibroblasts into myofibroblasts by suppressing oxidative stress via restoring the balance of Nrf2–NOX4. Nevertheless, some limitations of this study are as follows: the more details about the mechanism of anti-oxidative effect of JHF and the anti-fibrosis effect of various compounds contained in JHF still need to be proved with further investigations.

## Conclusion

This study indicated that JHF performed remarkably therapeutic and long-term effects on PF in rat and suppressed the differentiation of fibroblast into myofibroblast in MRC-5 cell through reducing the oxidative response by restoring the balance of Nrf2–NOX4. This work might present the details about anti-PF mechanisms of JHF and provide the basis for its widely clinical use.

## Author Contributions

YB, JL, PZ, and YL designed the outline of the study, contributed toward data analysis, and critically revised the paper. YB performed the experiments, conceived the study, wrote the draft, and revised the manuscript. ML, YQ, and YT were involved in performing the experiments, acquisition of data, and statistical analysis. SF and TZ contributed to the preparation of JHF. All authors read and approved the final manuscripts.

## Conflict of Interest Statement

The authors declare that the research was conducted in the absence of any commercial or financial relationships that could be construed as a potential conflict of interest.
